# Endothelial dysfunction: the early predictor of atherosclerosis

**DOI:** 10.5830/CVJA-2011-068

**Published:** 2012-05

**Authors:** Mashudu Mudau, Amanda Genis, Amanda Lochner, Hans Strijdom

**Affiliations:** Department of Biomedical Sciences, Division of Medical Physiology, Faculty of Health Sciences, Stellenbosch University, Stellenbosch, South Africa; Department of Biomedical Sciences, Division of Medical Physiology, Faculty of Health Sciences, Stellenbosch University, Stellenbosch, South Africa; Department of Biomedical Sciences, Division of Medical Physiology, Faculty of Health Sciences, Stellenbosch University, Stellenbosch, South Africa; Department of Biomedical Sciences, Division of Medical Physiology, Faculty of Health Sciences, Stellenbosch University, Stellenbosch, South Africa

**Keywords:** endothelium, endothelial dysfunction, nitric oxide bioavailability, eNOS uncoupling, oxidative stress, atherosclerosis

## Abstract

**Abstract:**

Since the discovery in the 1980s that nitric oxide (NO) is in fact the elusive endothelium-derived relaxing factor, it has become evident that NO is not only a major cardiovascular signalling molecule, but that changes in its bioavailability are crucial in determining whether atherosclerosis will develop or not. Sustained high levels of harmful circulating stimuli associated with cardiovascular risk factors such as diabetes mellitus elicit responses in endothelial cells that appear sequentially, namely endothelial cell activation and endothelial dysfunction (ED).

ED, characterised by reduced NO bioavailability, is now recognised by many as an early, reversible precursor of atherosclerosis. The pathogenesis of ED is multifactorial; however, oxidative stress appears to be the common underlying cellular mechanism in the ensuing loss of vaso-active, inflammatory, haemostatic and redox homeostasis in the body’s vascular system. The role of ED as a pathophysiological link between early endothelial cell changes associated with cardiovascular risk factors and the development of ischaemic heart disease is of importance to basic scientists and clinicians alike.

## Abstract

Between 1995 and 2004, cardiovascular diseases accounted for about 195 deaths per day in South Africa. Particularly disturbing is that cardiovascular mortality is expected to escalate by a staggering 41% in the working age group (35–64 years) in the South African population by the year 2030.^1^ In 2004, the World Health Organisation (WHO) reported cardiovascular diseases/ischaemic heart disease (IHD) to be the leading cause of death worldwide and that cardiovascular deaths are envisaged to escalate to 23.4 million by the year 2030.^2^

Atherosclerosis is a chronic progressive vascular disease, characterised by plaque formation and subsequent fissure, erosion or rupture of the plaque with thrombosis of the plaque surface.^3^ A complication of coronary atherosclerosis can be the development of myocardial ischaemia and ultimately myocardial infarction.^4^ Hypertension, tobacco use, dyslipidaemia, diabetes mellitus, physical inactivity and obesity, all of which are associated with the development of atherosclerosis and IHD, are considered to be the top risk factors for cardiovascular mortality worldwide.^2^

Chronic exposure to cardiovascular risk factors and the harmful circulating stimuli associated with these conditions overwhelms the defense mechanisms of the vascular endothelium, hence compromising its integrity and ultimately initiating endothelial dysfunction (ED).^5^ Mounting evidence is pointing to ED as one of the major pathophysiological links between exposure to cardiovascular risk factors and the development of atherosclerotic disease (Fig. 1).^6^

**Fig. 1.**
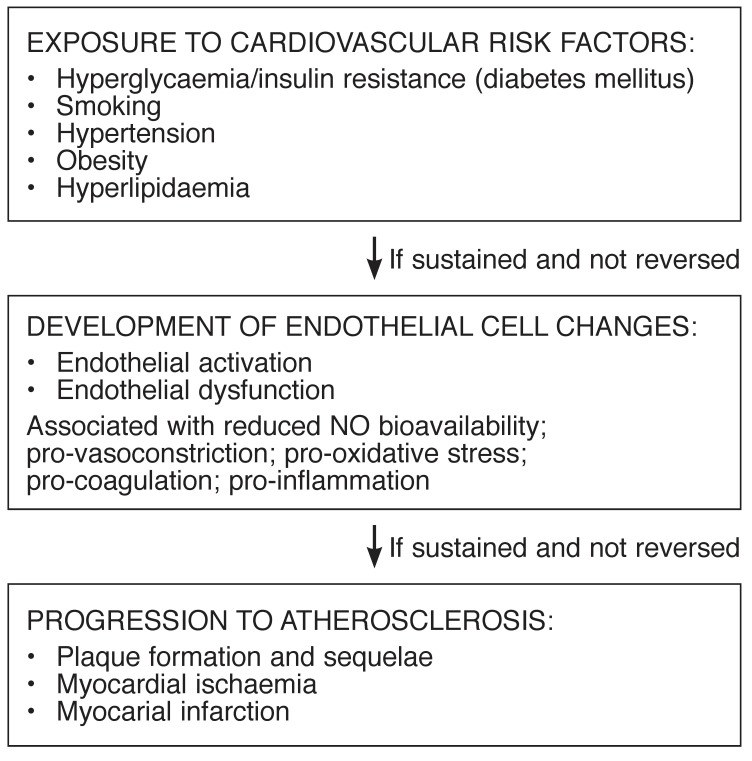
Exposure of endothelial cells to cardiovascular risk factors and the resultant pathophysiological changes, i.e. endothelial activation and dysfunction, with progression to atherosclerosis if risk-factor exposure is sustained.

ED is commonly associated with reduced nitric oxide (NO) bioavailability, and hence an inability of the endothelium to initiate vasodilation in response to vasodilatory stimuli such as acetylcholine or shear stress. It represents an initial reversible step in the development of atherogenesis, and for this reason, early clinical identification of ED may become an important tool in the prevention or reversal of progression to atherosclerosis and IHD.^7^

ED comprises a loss of balance between endothelial-derived vasodilatory and vasoconstrictory factors, where the pro-vasoconstrictory state becomes dominant, leading to progressive pathophysiological changes. These changes appear as sequentially occurring responses in endothelial cells, also referred to by some as the endothelial activation–dysfunction–injury triad.^8^,^9^ Collectively, these endothelial changes exhibit pro-inflammatory, pro-oxidant, proliferative, pro-coagulation and pro-vascular adhesion features.^7^,^10^

## The endothelium: a functional organ

The vascular endothelium consists of approximately 1–6 × 10^13^ endothelial cells and accounts for about 1 kg of total body weight. For many years after its discovery, the endothelium was believed to be an inert, semi-permeable barrier between circulating blood and the underlying sub-endothelial tissues.^11^ However, extensive research has since revealed a far more complex role for the endothelium. We now know that the endothelium is in fact a metabolically active organ, playing a crucial role in the maintenance of vascular homeostasis by releasing a variety of vasoactive factors that can either dilate or constrict the blood vessels, depending on the type of the stimulus.^7^

Vascular homeostasis entails keeping a tightly controlled balance between a vasodilatory state, which is often associated with anti-oxidant, anti-inflammatory and anti-thrombotic effects on one hand, and a vasoconstrictory state on the other, which is associated with pro-oxidant, pro-inflammatory and pro-thrombotic effects.^12^ The vasodilatory state is mediated by factors such as nitric oxide (NO), endothelium-derived hyperpolarising factor (EDHF) and prostacyclins, while a vasoconstrictory state is mediated by factors such as endothelin-1 (ET-1), angiotensin II and thromboxane A2.^7^,^12^ Of these endothelial-derived factors, NO, which was originally identified as the endothelial-derived relaxing factor (EDRF), has since evoked much interest as it is considered to be the most potent endogenously synthesised vasodilator in the body, and a key marker of endothelial function and dysfunction.

The vasoactive factors released by endothelial cells and their effects are summarised in Table 1. In view of the physiological, pathophysiological and clinical importance of NO in vascular physiology, we have included a brief discussion on the physiology of NO below.

**Table 1.** Overview Of Endothelium-Derived Vaso-Active Factors

*Endothelium-derived factors*	*Physiological effects*	*Enzymatic source and mechanism of action*
Nitric oxide (NO)	• Potent vasodilator	• Synthesised by the enzymes: eNOS, nNOS and iNOS, with eNOS the major endothelial source of NO during physiological conditions
	• Inhibits inflammation, VSMC proliferation and migration, platelet aggregation and adhesion, and leukocyte adhesion	
	• Regulates myocardial contractility	• Diffuses from endothelial cells to underlying VSMCs where it binds to soluble guanylyl cyclase, leading to a cascade of events that ultimately result in vascular relaxation
	• Regulates cardiac metabolism	
	• Cardioprotective during ischaemia–reperfusion injury	
Prostacyclin (PGI_2_)	• Vasodilatory agent	• Derived from arachidonic acid by cyclooxygenase-2 (COX-2)
	• Inhibits platelet aggregation	
Endothelium-derived hyperpolarising factor (EDHF)	• Exerts vasodilatory effects, particularly in small arteries of diameter ≤ 300 μm	• Its identity is still under suspicion with proposed candidates such as potassium ions and hydrogen peroxide
		• Causes relation of VSMCs by means of membrane hyperpolarisation
Endothelin-1 (ET-1)	• A potent vasoconstrictor	• Synthesised by endothelin-converting enzyme
		• Exerts its effects via two receptors: ET_A_ expressed on endothelial cells and ET_B_ on VSMCs. ET_A_ receptors promote vasoconstriction, whereas ET_B_ receptors promote NO production and ultimately reduction in ET-1 production
Thromboxane A (TXA_2_)	• A potent vasoconstrictor	• Derived from arachidonic acid by COX-1
Angiotensin ll	• A potent vasoconstrictor	• Synthesised by angiotensin converting enzyme
		• Elicits its effects via two receptors: AT_1_ which promotes vasoconstriction and cell proliferation, and AT_2_ which antagonises the effects of AT_1_

## Nitric oxide

The realisation in the 1980s that the identity of EDRF was in fact NO was rather astonishing, as NO had until then been perceived as nothing more than a toxic environmental pollutant found in cigarette smoke, exhaust fumes of motor cars and harmful gases generated by industrial processes.^13^,^14^ The ground-breaking discovery that NO is also synthesised in the body and functions as a chemical messenger with important physiological effects introduced a novel paradigm in cardiovascular physiology and pathophysiology. In addition to its vasodilatory properties, NO was also found to exert anti-inflammatory and cardioprotective effects.^15^

Owing to its gaseous and free-radical nature, NO is able to diffuse easily between cells and tissues and react with a variety of molecules in the body.^13^ NO is synthesised from the amino acid L-arginine by a family of enzymes known as nitric oxide synthase (NOS).^14^ The NOS enzyme occurs as three isoforms, namely neuronal NOS (nNOS), inducible NOS (iNOS) and endothelial NOS (eNOS).^14^,^16^,^17^ Physiologically, eNOS and nNOS are constitutive, calcium-dependent enzymes and continuously produce low levels of NO. On the other hand, iNOS is calcium independent, its expression is provoked by inflammatory cytokines, and it produces large amounts of NO, about 1 000-fold more than eNOS or nNOS.^13^ This can have potentially harmful consequences as excess NO can react with the free radical superoxide anion (O_2_–), yielding a harmful and highly reactive species, peroxynitrite.^13^

**Fig. 2.**
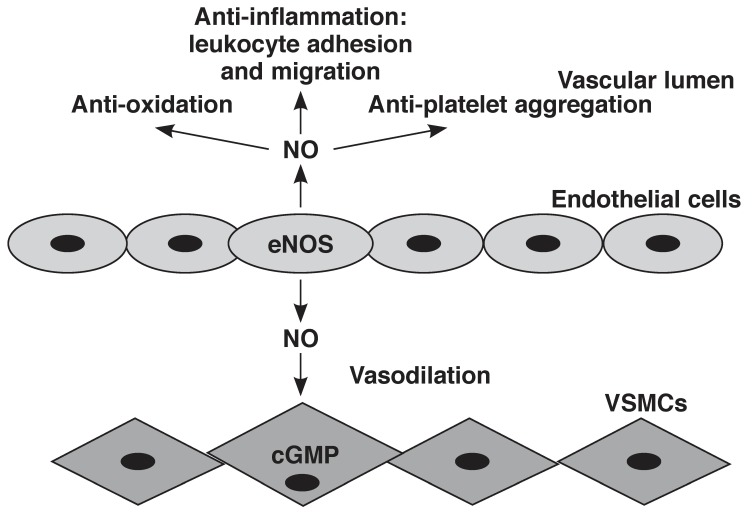
Synthesis of NO, downstream mechanisms and physiological effects. NO is synthesised by eNOS in the endothelial cells and diffuses into the underlying vascular smooth muscle cells (VSMCs), where it activates the second messenger, cyclic guanosine monophosphate (cGMP). Further downstream, signalling eventually leads to VSMC relaxation and vasodilation. In addition, NO regulates vascular homeostasis by anti-oxidation, anti-inflammatory and anti-platelet aggregation effects.

All NOS isoforms require cofactors such as (6R)-5,6,7,8-tetrahydrobiopterin (BH4), flavin adenine dinucleotide (FAD), flavin mononucleotide (FMN), and iron protoporphyrin IX (haem).^18^ Of the three isoforms, it has been proposed that eNOS is the major isoform responsible for NO production under physiological conditions in the cardiovascular system and endothelial cells in particular, leading to the classical signalling mechanism in the underlying vascular smooth muscle cells (VSMCs) and ultimately, relaxation^19^ (Fig. 2).

**Fig. 3.**
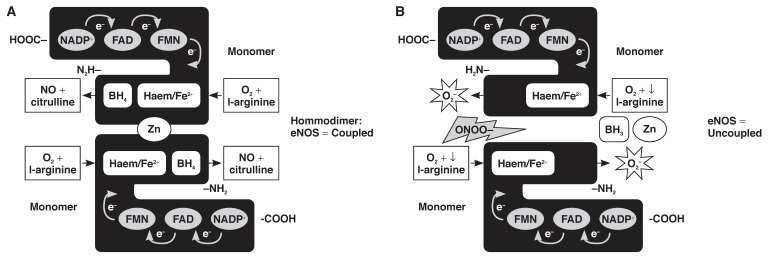
Coupled and uncoupled eNOS. (A) In the presence of sufficient levels of substrates and co-factors, and the absence of harmful reactive species, eNOS monomers will form a dimerised, coupled enzyme and produce physiological amounts of NO. (B) Decreased levels of the substrate, L-arginine and/or harmful effects exerted by increased levels of ONOO–, cause failure of the enzyme to dimerise, leading to the uncoupling of eNOS and the production of O_2_– instead of NO.

Failure of the eNOS protein to dimerise, or the absence of some of the cofactors mentioned above will lead to the enzyme catalysing the formation of O_2_– instead of NO, a mechanism referred to as eNOS uncoupling^20^ (Fig. 3). As will be discussed later, eNOS uncoupling is an important mediator of ED during a pathophysiological state.

## Cardiovascular risk factors associated with the development of ED

## Type 1 diabetes mellitus and insulin resistance/type 2 diabetes mellitus

Both type 1 and type 2 diabetes are independent risk factors for the development of accelerated atherosclerosis, IHD and cardiovascular disease in general.^11^ Similarly, type 1 diabetes mellitus, insulin resistance and type 2 diabetes mellitus have been shown to be strongly associated with the development of ED.^21^ In fact, the temporal progression from insulin resistance to type 2 diabetes mellitus has been postulated to be mirrored by the progression of ED to atherosclerosis.^22^

ED observed in diabetes mellitus is primarily attributable to (1) oxidative stress (increased O_2_– generation due to upregulated expression of NADPH oxidase), and (2) increased formation of advanced glycation end-products (AGEs).^23^,^24^ Aside from scavenging NO, causing decreased NO bioavailability and producing peroxynitrite, O_2_– also modifies the activity and regulation of eNOS, and promotes vascular smooth muscle cell (VSMC) proliferation and inflammation.^24^

Hyperglycaemia, as occurs in diabetes mellitus, results in non-enzymatic glycation of intracellular and extracellular proteins and lipids, which leads to the generation of AGEs. The latter subsequently accumulate in the vascular wall and reduce NO activity by quenching NO.^24^,^25^ AGEs also bind to specific surface receptors, called receptors for AGEs (RAGE), which are expressed on cells such as monocytes, macrophages and VSMCs, resulting in the amplification of an inflammatory response,^24^,^25^ increased vascular permeability and oxidative stress.^25^

Furthermore, hyperglycaemia is also known to activate protein kinase C (PKC), which decreases eNOS activity, leading to reduced NO and increased ET-1 production.^24^ In the setting of ED, ET_B_ receptor-mediated vasodilatory effects of ET-1 are blunted (refer to Table 1) and therefore the vasoconstrictory state predominates.^26^ PKC also enhances the expression of adhesion molecules such as ICAM, VCAM and E-selectin,^24^ which is associated with endothelial cell activation.

ED has been reported to occur early in insulin resistance.^22^ Often insulin resistance is associated with central adiposity and hence the metabolic syndrome, i.e. hypertriglyceridaemia, low high-density lipoprotein (HDL) levels, high low-density lipoprotein (LDL) levels and hypertension, all of which could potentially favour the development of ED and eventually atherogenesis.^22^

## Hyperlipidaemia

Hyperlipidaemia constitutes increased circulating lipids including cholesterol and triglycerides, a state which can predispose to ED. Possible mechanisms underlying hyperlipidaemia-induced ED include: (1) upregulation of NADPH oxidase, increased O_2_– production and oxidative stress, (2) increased plasma levels of asymmetric dimethylarginine (ADMA),^25^ and (3) oxidation of LDL.^27^ ADMA is an endogenous inhibitor of eNOS and competes with L-arginine for the same binding site on eNOS, thus resulting in eNOS uncoupling, increased O_2_– production and hence decreased NO production. Plasma concentrations of ADMA have been reported to be increased in hypercholesterolaemia,^28^,^29^ and this compound is considered to be both a marker and risk factor of ED.^28^

In addition to scavenging NO, excess O_2_– modifies LDL cholesterol to form oxidised LDL (ox-LDL), which plays a major role in the development of endothelial activation and atherogenesis.^30^ Ox-LDL has been reported to promote ET-1 production,^31^ expression of adhesion molecules and chemoattractants, as well as VSMC migration and proliferation.^27^ Furthermore, ox-LDL can be engulfed by macrophages forming foam cells which adhere to the vessel wall and contribute to the initiation of an atherosclerotic plaque.^27^

**Fig. 4.**
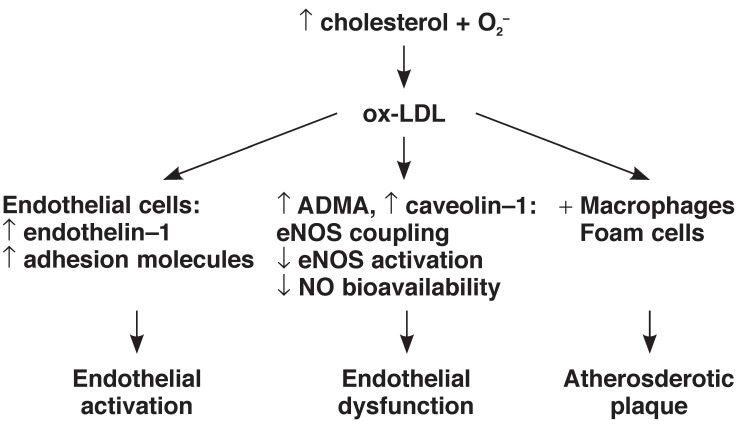
Pathophysiological effects and the interplay between increased plasma cholesterol and O_2_– levels, and endothelial cell responses.

Both LDL and ox-LDL have been shown to increase the activity of S-adenosylmethionine-dependent methyltransferases, which lead to increased ADMA synthesis. Therefore, LDL and ox-LDL may be accountable for the increased plasma levels of ADMA in hypercholesterolaemia.^32^ LDL or ox-LDL can also upregulate caveolin-1 synthesis and thus inhibit eNOS activity^33^,^34^ (Fig. 4).

## Hypertension

ED is a prominent underlying feature of hypertension,^35^ and patients with hypertension have been shown to demonstrate blunted forearm blood flow in response to vasodilatory stimuli such as acetylcholine and bradykinin,^36^ which is indicative of ED. Increased production of ROS and endothelial-derived contracting factors (EDCFs) such as ET-1, angiotensin II, PGH2 and TXA_2_, and decreased NO bioavailability are all observed in patients with hypertension.^26^,^36^

Shear stress is known to be one of the most important mechanisms of inducing NO-mediated vasodilation in both the micro- and macrovasculature. However, this response is reduced or absent in hypertensive patients.^37^ In addition to this, Iaccarino *et al*.^38^ observed decreased protein kinase B (PKB)/Akt-dependent activation of eNOS in a model of spontaneously hypertensive rats (SHR).

In a recent study, the role of oxidative stress and ED in the development of hypertension in spontaneously hypertensive rates was investigated.^35^ The results showed that early treatment with the antioxidant reservatrol was associated with reduced oxidative stress markers, improved endothelium-dependent vasodilatation and an attenuation in the development of hypertension in these animals.

## Smoking

Tobacco smokers exhibit decreased NO bioavailability, increased levels of ox-LDL, and impaired flow-mediated vasodilation, phenomena which are all highly suggestive of ED.^39^ Passive smoking has recently also been implicated in impairment of endothelial function.^39^,^40^ It appears that the harmful effects of smoking on endothelial cells are dose dependent and reversible upon smoking cessation.^39^ As with other cardiovascular disease risk factors, oxidative stress appears to be the major mechanistic link between smoking and ED.^39^,^41^

Cigarette smoke is rich in free radicals and directly delivers free radicals to the body. Besides being the supplier of free radicals, cigarette smoke facilitates endogenous release of ROS via activation of inflammatory cells.^41^,^42^ Furthermore, smoking has been reported to decrease the levels of HDL cholesterol, which is known to have anti-endothelial dysfunction and anti-atherosclerotic properties.^43^

## Aging

Increasing age has been recognised as one of the factors that predisposes to ED.^43^,^44^ With aging, the ability of the endothelium to produce NO is reduced.^45^ Furthermore, some studies have reported reduced expression and activity of eNOS as well as decreased expression of a major downstream target molecule of NO, soluble guanylyl cyclase (sGC) in VSMCs, and its activity in older animals.^45^ In addition to the decreased NO production, other endothelial-derived relaxing factors (EDRFs) (prostacyclin and EDHF) are also reduced, while endothelial-derived contracting factors (EDCFs) such as ET-1 and COX-derived prostanoids, and ROS production are increased.^44^,^45^ Plasma levels of ADMA are also known to rise with increased age.^45^

One of the mechanisms contributing to reduced NO levels in aging may be the increased activity of arginase I.^43^,^44^ Arginase I is an enzyme that catalyses conversion of L-arginine to L-ornithine and urea, and it thus competes with eNOS for L-arginine.^43^ Hence, the increased activity of this enzyme as observed with advancing age may result in uncoupling of eNOS, reduced NO production and hence ED.^43^,^44^ Clearly the balance between EDRFs and EDCFs is lost with advancing age, establishing aging as a risk factor for the development of ED. Moreover, aging is often associated with co-morbid conditions such as diabetes, hypertension and hypercholesterolaemia, further exacerbating the risk of developing ED, atherosclerosis and ultimately cardiovascular diseases.^44^

## Proposed mechanisms of ED

Oxidative stress appears to be the common underlying cellular mechanism for the development of ED in all the risk factors discussed above. According to the literature, cardiovascular risk factors are associated with upregulation of ROS sources, especially NADPH oxidase.^7^,^20^ However, other sources of ROS such as xanthine oxidase, cyclooxygenase (COX) and mitochondria also play a role.^23^ In fact, eNOS *per se* becomes a potential ROS generator when in the uncoupled state.^20^ Harmful effects of oxidative stress include increasing VSMC proliferation (resulting in thickening of the vascular wall), endothelial cell apoptosis, and increased expression and activity of matrix metalloproteinases, which are involved in the establishment of an atherosclerotic plaque.^39^

Oxidative stress comprises increased rates of oxidant production and decreased levels of antioxidant activity [e.g. superoxide dismutase (SOD), vitamin C and E, etc.].^46^ Under physiological conditions, the enzyme SOD regulates the levels of O_2_–.^47^ However, increased generation of O_2_– overwhelms the defensive mechanisms of SOD, leaving O_2_– free to react with other molecules, particularly NO, for which it has a greater affinity.^47^

**Fig. 5.**
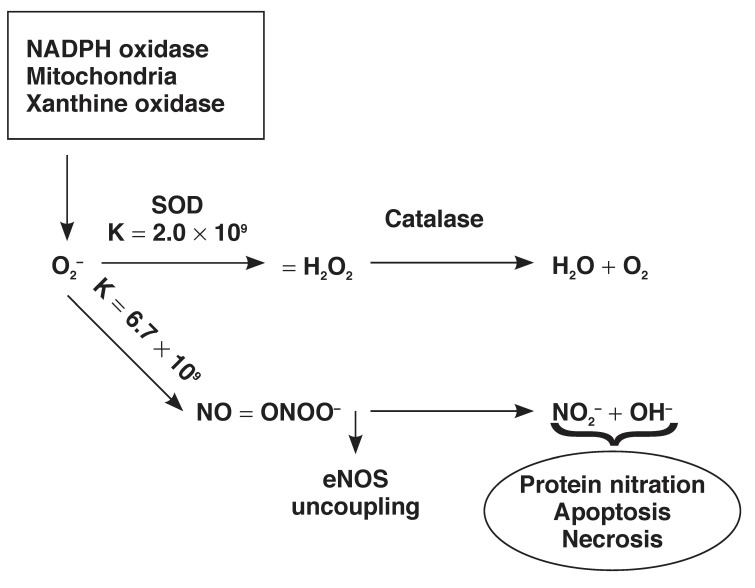
Oxidative and nitro-oxidative stress. Superoxide anion (O_2_–) released from sources such as NADPH oxidase, mitochondria and xanthine oxidase is dismutated to hydrogen peroxide (H_2_O_2_) by superoxide dismutase (SOD), which is then converted to water and oxygen by catalase. However, O_2_– has a higher affinity for NO than SOD, and when in excess, it preferentially combines with NO to produce peroxynitrite with various pathophysiological consequences.

O_2_– is implicated in the direct induction of ED by the scavenging of NO, leading to the production of the highly reactive and harmful reactive nitrogen species (RNS), peroxynitrite.^48^ In fact, the reaction between O_2_– and NO has been reported to occur much faster (rate constant = 6.7 × 10^9^ m/s) than that of dismutation of O_2_– by SOD (rate constant = 2.0 × 10^9^ m/s).^49^ High levels of peroxynitrite are injurious to the cells, oxidatively damaging DNA, lipids and proteins. In addition to being cytotoxic, peroxynitrite damages the intricate eNOS structure, leading to eNOS uncoupling, which further perpetuates the ED vicious circle^50^ (Fig. 5).

Peroxynitrite has been reported to oxidise the essential cofactor of eNOS, BH_4_ to its inactive form, trihydrobiopterin radical (BH_3_–), which in turn leads to uncoupling of eNOS.^20^,^50^,^51^ Furthermore, peroxynitrite may oxidise the zinc thiolate cluster in the centre of the eNOS enzyme, resulting in the loss of the zinc ion and the formation of disulfide bonds between the enzyme monomers, and thus disruption of the binding site for BH_4_ and L-arginine^20^,^52^ (Fig. 3). Vitamin C is able to recycle BH_3_– to BH_4_,^50^,^51^ and supplementation with BH_4_ has been reported to restore endothelial function in conditions such as insulin resistance, hypercholesterolaemia,^51^ diabetes mellitus and essential hypertension, as well as in chronic smokers.^20^

In addition to peroxynitrite-induced eNOS uncoupling, other oxidants such as hydrogen peroxide have also been shown to uncouple the enzyme. Therefore, during conditions of oxidative stress, eNOS deviates from its role of being an essential regulator of the functioning of the cardiovascular system to being an O_2_– releasing enzyme. A vicious circle therefore develops, whereby uncoupled eNOS synthesises O_2_– at the expense of NO, further aggravating oxidative stress.

Inflammation is another common underlying mechanism of ED.^53^ Under physiological conditions, the endothelium regulates vascular inflammation (including expression of adhesion molecules and leukocyte adhesion) via the release of NO.^54^ It is therefore more likely that ED will promote sustained vascular inflammation, which is detrimental to the vascular system. However, several studies have reported that inflammation also promotes ED and it is therefore recognised as a novel risk factor for cardiovascular diseases.^53^,^55^

There seems to be a causal relationship between oxidative stress and inflammation. Oxidative stress may amplify vascular inflammation signalling pathways,^56^ and conversely inflammatory cells increasingly release O_2_–. Inflammation is often associated with the overexpression of inflammatory cytokines such as tumour necrosis factor-alpha (TNF-α) and interleukin-1 (IL-1). These inflammatory cytokines in turn prompt endothelial cells or macrophages to express adhesion molecules such as VCAM-1 and ICAM-1, MCP-1, interleukin-6 (IL-6) resulting in a state of endothelial activation, which is a precursor of ED^57^ (Fig. 1).

The role of TNF-α in ED has received considerable attention in recent years, and is now well appreciated. High levels of TNF-α have been associated with cardiovascular diseases such as acute myocardial infarction, chronic heart failure, atherosclerosis and myocarditis.^58^ Increased TNF-α levels are also significantly correlated with obesity, which is an independent risk factor for ED.^59^ This inflammatory cytokine has been reported to promote ROS formation via NADPH oxidase and xanthine oxidase.^60^ For example, Gao *et al*. reported that TNF-α induces ED via increased NADPH oxidase activity in coronary arterioles of mice with type 2 diabetes.^61^ In addition, TNF-α has been implicated in the downregulation of eNOS expression (and therefore decreased NO production) by accelerating eNOS mRNA degradation.^34^,^60^,^62^ According to Zhang *et al*., ED observed in myocardial ischaemia–reperfusion injury may be attributable to increased TNF-α expression via the enhancement of xanthine oxidase activity.^63^

Other harmful stimuli-induced mechanisms associated with the development of increased oxidative stress and decreased eNOS activation/eNOS uncoupling include activation of cholesterylester transport protein (CETP), downregulation of lipoprotein lipase, downregulation of peroxisome-proliferator activated receptor (PPAR), downregulation of protein kinase A (PKA), activation of caveolin, activation of rho-kinase and downregulation of sphingosine-1-phosphate.^64^

## Assessment of endothelial function

## Direct mechanical endothelial function measurements

Direct endothelial function measurement in humans has the potential to become an important clinical tool in diagnosing or predicting the development of cardiovascular disease in the presence or absence of cardiovascular risk factors. Furthermore, in recognition of evidence pointing towards a pathophysiological link between ED and IHD, a number of human experiments have been conducted in which clinical assessment of ED is explored as a possible predictor or prognostic marker of cardiovascular events.^65^

An ideal method for the direct measurement of endothelial function should be safe, cost-effective, non-invasive, repeatable, reproducible and standardised between laboratories.^5^,^65^ Current methods of assessing endothelial function include flow-mediated dilation (FMD), forearm plethysmography, finger-pulse plethysmography, pulse curve analysis and quantitative coronary angiography^65^ (Table 2).

**Table 2.** Clinical Detection Techniques Of Endothelial Function

*Method*	*Brief description*
Forearm plethysmography	Involves intrabrachial infusion of endothelial-dependent vasodilators such as acetylcholine, metacholine, substance P and bradykinin, with subsequent measurement of changes in endothelial function of forearm arterioles
Flow-dependent dilation of the brachial artery	This method employs a high-resolution ultrasound to quantify flow-mediated dilation of the brachial artery
Finger-pulse plethysmography (ENDO-PAT)	A novel non-invasive technique that measures changes of the pulse-wave amplitude during reactive hyperaemia. Low pulse-wave amplitudes are associated with compromised endothelial function and are therefore good predictors of cardiovascular disease
Pulse curve analysis	A non-invasive technique that relies on the measure of arterial stiffness to quantify endothelial function
Quantitative coronary angiography following intracoronary infusion of acetylcholine	An invasive approach of quantifying endothelial function, which involves intracoronary infusion of the endothelium-dependent vasodilator, acetylcholine, and subsequent measurment of the vasomotor response

In a recent review article, FMD was recognised as a commonly undertaken, non-invasive technique to assess endothelial function.^66^ In this article, a meta-analysis of 14 studies (more than 8 300 subjects) showed that FMD was strongly predictive of future cardiovascular events. Despite the evidence in favour of the independent prognostic value of vascular/endothelial function measurements, the authors conceded that more and larger human studies should be undertaken to confirm this finding.

## Biomarkers

Although many biomarkers of endothelial function have been identified, only some may have potential clinical use. Therefore, the measurement of endothelial biomarkers remains a tool mainly utilised in the experimental animal and *in vitro* laboratory setting.

## Reduction of NO bioavailability

Reduction in endothelial-derived NO production or bioavailability represents a measurable parameter that is suggestive of the development of ED. Laboratory-based studies most often make use of indirect NO measurements (measurements of metabolic products of NO) to confirm ED, such as nitrogen oxide levels^67^ or levels of stable degradation products, nitrite and nitrate.^68^ Many researchers also measure expression and/or activation of eNOS, the main enzymatic source of NO in endothelial cells, to further validate their findings.^69^,^70^

In humans, changes in NO production can also be detected in plasma by measurement of NO metabolites. Kiettisanpipop *et al*. reported a decrease in plasma NO metabolite levels in patients with severe pulmonary hypertension compared to those with moderate hypertension.^71^ Oestrogen replacement therapy has been shown to increase plasma NO levels while decreasing ET-1 levels and thus improving endothelial function in postmenopausal women.^72^ Heiss *et al*. demonstrated that plasma levels of NO derivatives (nitrosyl/nitroso species) were decreased in patients with endothelial dysfunction.^73^ It remains to be seen, however, whether the measurement of plasma NO or NO-derived metabolites will become a widely used clinical tool with predictive properties.

## ADMA

ADMA has emerged as a mediator, independent risk factor and, from a clinical perspective, potentially promising marker of ED.^29^ As explained earlier, ADMA is endogenously synthesised via methylation of arginine residues in the nuclear proteins^74^ and competitively inhibits eNOS, resulting in decreased NO production, which may induce eNOS uncoupling. Synthesis of ADMA is catalysed by protein arginine methyltransferases (PRMTs) and its degradation is catalysed by dimethylarginine dimethylaminohydrolases (DDAH).^75^

DDAH levels are often decreased in a variety of cardiovascular diseases, which leads to the upregulation of ADMA. For example, treatment of endothelial cells with TNF-α, ox-LDL and glucose has been reported to diminish the activity of DDAH.^74^,^75^ Furthermore, PRMTs and DDAH are ROS-sensitive; the activity of the DDAH is impaired, whereas the activity of the PRMTs is increased in conditions of oxidative stress.^75^

Indeed, increased plasma levels of ADMA have been documented in patients with conditions such as hyperlipidaemia, hypertension, coronary artery disease, stroke and end-stage renal disease.^75^ Furthermore, ADMA was found to be significantly elevated in patients with unstable angina, and reduced plasma levels of ADMA at six weeks post-percutaneous coronary intervention was found to be possibly indicative of a reduced risk of recurrent cardiovascular events.^76^

A recent study investigated the prognostic value of ADMA with regard to cardiovascular disease and death (fatal or non-fatal myocardial infarction, coronary insufficiency, angina pectoris, stroke or TIA, intermittent claudication or heart failure) in Framingham Offspring study participants.^77^ Although ADMA was significantly associated with all-cause mortality in this population, the study could not find an association between ADMA and cardiovascular disease incidence.

## Circulating endothelial cells and endothelial microparticles

Circulating endothelial cells (CECs), which are mature cells that have detached from the endothelium, represent a novel biomarker of endothelial injury.^78^,^79^ In a healthy person, the endothelium is constantly refurbished at a replication rate of < 1% and levels of CECs are very low. Studies using a flow-activated cell sorter (FACS) isolation technique reported CECs ranging from 50–7 900 cells/ml in healthy individuals and up to 39 100 cells/ml in individuals with vascular diseases.^79^

Potential mechanisms underlying endothelial cell detachment may be mechanical injury, action of proteases and/or cytokines, defective endothelial cell adhesion to the extracellular matrix, cellular apoptosis, and injurious actions of cardiovascular risk factors, such as occur during the induction of ED. Increased levels of CECs are associated with ED, cardiovascular diseases and a variety of other diseases.^78^,^79^ Apoptotic CECs expressing surface marker (CD146) have been reported to be increased in patients with cardiovascular disease.^65^

Other circulating cellular markers of endothelial injury include endothelial microparticles (EMPs), which are small cell membrane vesicles released into the circulation by activated or apoptotic cells.^5^,^65^ Patients with hypertension and coronary artery disease have been reported to demonstrate high levels of EMPs,^65^ and according to Tramontano *et al*., statins can diminish the release of EMPs in cultured coronary endothelial cells.

## Nitrotyrosine upregulation

In addition to its ability to directly uncouple the eNOS enzyme, which can lead to ED, peroxynitrite undergoes protonation to form peroxynitrous acid (ONOOH), or it can combine with carbon dioxide (CO_2_) to form nitroso-peroxocarboxylate (ONOOCO_2_–), both of which yield tyrosine-nitrating compounds.^80^,^81^ Via formation of these compounds, peroxynitrite leads to nitration (addition of a NO_2_ group) of tyrosine residues of proteins, leading to formation of nitrotyrosine.^82^

Under normal conditions, low levels of free or protein-bound nitrotyrosine are detectable, which may indicate low levels of oxidants and nitrating species produced during physiological processes. However, significant nitrotyrosine upregulation is observed in conditions that are associated with nitroxidative stress such as inflammation, cardiovascular disease (including ED and atherosclerosis) and neurodegenerative disorders.^80^ Tyrosine nitration may modify the structure and function of proteins, leading to alterations in catalytic activity of enzymes, production of antigenic epitopes, and impaired cell signal transduction.^82^

It has recently been proposed that nitrotyrosine levels can be clinically detected in urine samples using a surface plasmon resonance (SPR) sensor^83^ or high-performance liquid chromatography.^84^ However, nitrotyrosine measurements in the context of ED research remain confined to the experimental laboratory setting.

## Other biomarkers of ED/vascular injury

Recently, the European Society of Cardiology Working Group on Peripheral Circulation published a position statement on methods for evaluating endothelial function.^85^ In this comprehensive review, several biochemical markers and assays that are used to examine different aspects of endothelial function were discussed. The working group mooted plasma ADMA levels as a potential biomarker of endothelial function; however the authors cautioned that currently, direct endothelial function measurements remain a superior indicator and should not be replaced by plasma ADMA level measurements due to inconsistent prognostic data obtained with the latter.

Another biomarker with potential clinical application is oxidised LDL levels; however this biomarker also presents with some limitations. It is difficult to determine ox-LDL levels *in vivo* and the ability of elevated plasma ox-LDL to independently predict the development of coronary heart disease is still equivocal.

In a recent study on a model of rat carotid injury, proteomic analysis of blood proteins showed significantly differential expression of vitamin D binding protein (VDBP), aldolase A (aldo A) and apolipoprotein E (ApoE) two weeks after injury.^86^ Reduced circulating levels of all three of these plasma markers were associated with the presence of vascular injury and may represent novel markers of ED; however, further research is necessary.

## Summary of assessment of endothelial function

With the development of an ever-increasing number of measurement techniques of endothelial function (both direct mechanical endothelial function assessment and measurement of biomarkers of endothelial function), most authors agree that more and larger human-based studies are necessary to validate their clinical usefulness. The overall objective of such studies should ultimately be to establish standardised protocols allowing for the clinical diagnosis of ED, and quantification of cardiovascular risk, followed by the reversal of ED by means of anti-ED therapies. We are not there yet. The various endothelial function assessment tools at our disposal should be compared in studies that account for, among others, pathophysiological relevance and reproducibility, predictability in diverse patient populations, ease of use, cost-effectiveness, and risk assessment abilities that are superior to the tools currently in use.^85^

Another shortcoming in our understanding of the clinical significance of ED is the lack of studies where the effects of therapies that specifically target endothelial biology are investigated. In this regard, promising observations were made in a study which showed that dietary supplementation with the NO-donor, L-arginine significantly improved endotheliumdependent dilatation in young adults with hypercholesterolaemia.^87^ Similarly, in another study on rabbits, dietary supplementation with L-arginine prevented hypercholesterolaemia-induced ED by augmentation of NO-production.^88^

## Progression of ED to atherosclerosis

ED has emerged as a potentially valuable prognostic tool in predicting the development of atherosclerosis and ultimately IHD.^89^ The progression from the early changes observed in compromised vascular endothelium (endothelial activation and dysfunction) to atherosclerosis is complex and multifactorial.^89^ The healthy, intact endothelium is a highly selectively permeable barrier and does not promote leukocyte adhesion and invasion, or platelet aggregation and adhesion.^53^ However, as the endothelium progresses to a dysfunctional state, vascular homeostasis becomes impaired, leading to reduced anti-oxidant, anti-inflammatory and anti-thrombotic properties (due to reduced NO bioavailability), enhanced endothelial permeability (barrier dysfunction), upregulated pro-inflammatory cytokine levels, and expression of adhesion molecules such as VCAM-1 and ICAM-1, which facilitate leukocyte adhesion to the endothelium.^53^

Leukocyte adhesion represents one of the first steps in the initiation of atherosclerosis. After adhering to the endothelium, leukocytes (monocytes and lymphocytes) cross the endothelium and migrate into the intima.^54^,^90^ Migration to the intima is mediated by chemo-attractants such as monocyte chemotactic protein-1 (MCP-1).^91^ Upon reaching the intima, monocytes transform into macrophages and express receptors that facilitate uptake of lipids. Uptake and accumulation of lipids lead to the transformation of macrophages into foam cells, which initiate an atherosclerotic lesion and further enhance release of inflammatory cytokines.^54^,^90^ Through these complex mechanisms, a cascade of events, which begins with the formation of an early atherosclerotic lesion, leading to an advanced lesion characterised by a plaque formation ensues.^90^

## Anti-endothelial dysfunction therapies

It has been shown that interventions such as lifestyle modification (exercise and diet) and various classes of pharmacological drugs can improve endothelial function, and in some instances reduce the risk of cardiovascular diseases.^22^,^92^ The anti-ED properties of statins have been extensively studied. Statins such as pravastatin, atorvastatin, simvastatin and fluvastin have all been shown to play an important role in correction of ED by improving endothelium-dependent vasodilation, in addition to their plasma cholesterol-lowering effects.^33^

Indeed, statin-induced eNOS activating effects have been demonstrated in investigations where addition of an eNOS inhibitor (L-NMMA) to pravastatin-treated hypercholesterolaemic patients hampered the endothelium-dependent vasodilation.^93^ Moreover, statins have been reported to stimulate activity of PKB/Akt, a major upstream activating signalling molecule of eNOS, and increase stability of eNOS mRNA, thus enhancing eNOS expression.^33^

In addition to statins and NO donors (discussed above), other drugs that have been shown to improve endothelial function include angiotensin converting enzyme (ACE) inhibitors, angiotensin receptor (AT1 receptor) blockers, peroxisome proliferator-activated receptor-γ (PPAR-γ) agonists, antioxidants and oestrogen replacement.^22^,^92^ In a recent review article, Balakumar *et al*. identified potentially novel target sites for the pharmacological improvement of vascular function.^64^ These target sites include rho-kinase, poly (ADP ribose) polymerase, protein tyrosine phosphatase (PTPase), Akt, protein kinase A (PKA), caveolin, cholesterylester transfer protein (CETP), lipoprotein lipase, sphingosine-1-phosphate (S1P), advanced glycation end-product (AGE) and transketolase, geranylgeranyltransferase (GGT), epoxide hydrolase and Janus kinase (JAK).

## Conclusion

In view of the ever-increasing prevalence of ischaemic heart disease in the developed and developing world, it has become imperative to identify and investigate mechanisms of early, potentially reversible pre-atherosclerotic changes in the endothelium. To date, the most clearly defined and well-understood early precursor of atherosclerosis is ED. In fact ED can be regarded as the *primum movens* of atherosclerotic disease. Several cellular mechanisms and markers of ED that could potentially lead to the development of early detection and therapeutic interventions have been determined. However, more research aiming at improving our understanding of ED is necessary in order to establish its detection and reversal as essential and routinely utilised future tools in the prevention of IHD.
